# Transport Patterns and Potential Sources of Atmospheric Pollution during the XXIV Olympic Winter Games Period

**DOI:** 10.1007/s00376-022-1463-1

**Published:** 2022-04-06

**Authors:** Yuting Zhang, Xiaole Pan, Yu Tian, Hang Liu, Xueshun Chen, Baozhu Ge, Zhe Wang, Xiao Tang, Shandong Lei, Weijie Yao, Yuanzhe Ren, Yongli Tian, Jie Li, Pingqing Fu, Jinyuan Xin, Yele Sun, Junji Cao, Zifa Wang

**Affiliations:** 1 State Key Laboratory of Atmospheric Boundary Layer Physics and Atmospheric Chemistry, Institute of Atmospheric Physics, Chinese Academy of Sciences, Beijing, 100029 China; 2 College of Earth and Planetary Sciences, University of Chinese Academy of Sciences, Beijing, 100049 China; 3 Center for Excellence in Regional Atmospheric Environment, Institute of Urban Environment, Chinese Academy of Sciences, Xiamen, 361021 China; 4 Inner Mongolia Autonomous Region environmental monitoring central station, Hohhot, 010090 China; 5 Institute of Surface-Earth System Science, Tianjin University, Tianjin, 300072 China; 6 Collaborative Innovation Center on Forecast and Evaluation of Meteorological Disasters, Nanjing University of Information Science and Technology, Nanjing, 210044 China; 7 Institute of Atmospheric Physics, Chinese Academy of Sciences, Beijing, 100029 China

**Keywords:** Olympic Winter Games, FLEXPART, transport characteristics, atmospheric pollution sources, 北京冬奥会, FLEXPART, 大气传输特征, 潜在污染源

## Abstract

The attainment of suitable ambient air quality standards is a matter of great concern for successfully hosting the XXIV Olympic Winter Games (OWG). Transport patterns and potential sources of pollutants in Zhangjiakou (ZJK) were investigated using pollutant monitoring datasets and a dispersion model. The PM_2.5_ concentration during February in ZJK has increased slightly (28%) from 2018 to 2021, mostly owing to the shift of main potential source regions of west-central Inner Mongolia and Mongolian areas (2015–18) to the North China Plain and northern Shanxi Province (NCPS) after 2018. Using CO as an indicator, the relative contributions of the different regions to the receptor site (ZJK) were evaluated based on the source-receptor-relationship method (SRR) and an emission inventory. We found that the relative contribution of pollutants from NCPS increased from 33% to 68% during 2019–21. Central Inner Mongolia (CIM) also has an important impact on ZJK under unfavorable weather conditions. This study demonstrated that the effect of pollution control measures in the NCPS and CIM should be strengthened to ensure that the air quality meets the standard during the XXIV OWG.

**Electronic supplementary material:**

Supplementary material is available in the online version of this article at 10.1007/s00376-022-1463-1.

## Abstract (zh)

PM_2.5_(环境空气中空气动力学当量直径小于等于2.5微米的颗粒物)在大气中的滞留时间长, 可传输至几百公里远. 某一城市或区域的大气PM2.5污染往往具有明显的区域性和复合型污染特征, 既包括本地排放生成, 也包括周边地区的外来传输贡献. 因此确保环境空气质量达标, 对成功举办第24届北京冬季奥林匹克运动会至关重要. 为了解区域大气污染物的传输规律和潜在来源, 我们利用历史大气污染物数据集、 大气传输模式和中国多分辨率排放清单, 对冬奥会期间比赛区域空气质量水平、 大气传输规律和潜在污染源贡献进行了研究. 根据轨迹聚类和源-受体扩散模型模拟分析, 我们发现影响张家口地区的气团来源存在系统性变化, 主要传输路径在2018年后明显从内蒙古中部城市群和蒙古国地区转移到华北平原和山西省北部, 气团的传输高度主要集中在距地面600米以下. 以一氧化碳为示踪剂, 根据“源-受体”关系法和排放清单, 我们评估了不同地区对受体站点张家口的相对贡献. 2018至2021年, 来自华北地区污染的相对贡献率从35%增加到68%, 而来自内蒙古中部污染的相对贡献率从56%下降到15%. 模拟的结果与观测值之间的相关系数达到约0.94, 且与MOPITT卫星数据较为一致.

**Article Highlights:**

- The sources of air mass affecting ZJK have a systematic shifting based on the meteorological datasets from 2015 to 2021.
- The air pollution from NCPS had a greater influence on the air quality of ZJK than CIM during the OWG.
- Reducing emissions in NCPS will play a crucial role in ensuring air quality for the OWG.

### 1. Introduction

The XXIV Olympic Winter Games (OWG) was held in Beijing (BJ) and Zhangjiakou (ZJK), Hebei Province, China, from 4 to 20 February 2022. The air quality on the North China Plain (NCP) was of particular interest both nationally and internationally. During recent years, the challenges associated with particulate pollution in China has received much attention, and corresponding measures have had a positive effect on improving air quality in the NCP (Xiong et al., 2017; Guo et al., 2020); however, particulate matter pollution due to regional transport remains a very serious issue (Khuzestani et al., 2017; Chang et al., 2019; Chen et al., 2021a;

Zhang et al., 2021a, b). Additionally, with the frequent occurrence of windblown sand and dust in the winter and spring, the air quality problem in the NCP requires sizable attention (Ren et al., 2004; Tian et al., 2018, 2020; Liang et al., 2020). The pollutants, $SO_2$, $NO_x$, and VOCs, related to fuel-burning from power plants, industry, domestic heating, and vehicle exhaust, may strongly influence the air quality of the downwind areas of the NCP (Hao et al., 2005; Ge et al., 2012). The enhancement of southwesterly and southeasterly winds usually causes surface pollution in BJ during the winter (Hua et al., 2017; Liu et al., 2020; Li et al., 2021c). Under the control of an anticyclone, pollutants can hover in Beijing as a consequence of local winds causing heavy regional pollution (Wang et al., 2007; Wei et al., 2012). Through a combination of observations and modeling, a previous study also found that 20% of $PM_{10}$ and 23% of $SO_2$ in Beijing air originated from the southern areas of BJ (Zhang et al., 2004; Li et al., 2017a). The transport and accumulation of air pollutants from south to north affects the air quality of OWG cities.

It is difficult to quantify the regional transport of pollutants through single observations; thus, numerical air quality transport models have become an important tool for studying the regional transport of pollutants (Li et al., 2008, 2013; Ge et al., 2012). Currently, the FLEXPART model has been used extensively to study the long-range transport of tracers in the atmosphere and to invert inventories of halogenated greenhouse gas emissions sources (Stohl et al., 2009; Li et al., 2010; Brioude et al., 2013; Pan et al., 2014; Long et al., 2019; Rigby et al., 2019). The FLEXPART model has been previously used to study the dispersion of carbon monoxide (CO) from the source regions of forest fires or wildfires to remote places (Warneke et al., 2006; Thakur et al., 2019). The FLEXPART model was adopted to simulate the atmospheric dispersion of radioactive materials emitted from the Fukushima nuclear power plant into the tropical western Pacific and Southeast Asia (Long et al., 2019). The combination of FLEXPART and emission inventory can accurately simulate the CO concentration of a specific site and trace the clean and polluted sources to the observation station (Li et al., 2010; An et al., 2014; Pan et al., 2014). Carbon monoxide (CO) is mainly a product of incomplete combustion, and anthropogenic emissions of CO account for more than 50% of total CO emissions (Duncan et al., 2007). The residence time of CO in the atmosphere is on the order of weeks to months and is a good tracer for studying atmospheric transport (Pan et al., 2014).

Air quality assurance during the Olympic Games is critical to its smooth operation (Wang et al., 2010; Yang et al., 2010). There are few studies on the atmospheric transport characteristics during the same period of the XXIV OWG, which is a very important reference for air quality assurance during the XXIV OWG. In this study, we used a combination of models and observations to study the historical pollution conditions, transport characteristics, and sources at OWG sites during the same period of the XXIV OWG. We chose CO as a tracer and used the FLEXPART model to simulate the 10–day backward footprint of 2015–21 during the same period of the XXIV OWG in ZJK (40°–42°N, 114°–116°E). Statistical analysis of the pollutant concentrations and horizontal and vertical trajectories for different years reveals the transport pathways and distribution of potential emission source areas of atmospheric particulate matter for OWG sites and their surrounding areas. This effort helped to protect air quality during the XXIV OWG by taking control measures in potentially polluted areas around the OWG sites before the start of the XXIV OWG.

The remainder of this paper is organized as follows. Section 2 presents the materials, methods, and details of the model used in this study. Discussions and Results are presented in section 3. Section 4 summarizes the results and presents concluding remarks.

### 2. Materials and methods

#### 2.1. *Sampling and atmospheric observations*

Two sites in BJ (116.4°E, 39.98°N) and ZJK (114.904° E, 40.8725°N) were selected to study the atmospheric pollution conditions in OWG locations for the period of 2015–21 (Fig. 1b). The emission of CO was relatively high at these two sites. Figure 2 shows the bivariate polar plots of $PM_{2.5}$, CO, $NO_2$, and $SO_2$ during the XXIV OWG for the 2015–21 period in ZJK. In general, higher concentrations of pollutants occurred in the region with low wind speeds ( $< 4$ m $s^{-1}$) from the east. Moderately-high concentrations of $PM_{2.5}$, CO, and $NO_2$ occurred in the region with high wind speeds ( $> 5$ m $s^{-1}$) from the northwest. Local emissions and eastward transport of pollutants should be considered. The China Environmental Monitoring Centre real-time monitoring data is the source of air quality data used in this study (hourly average values in February) (http://113.108.142.147:20035/emcpublish, last access: 24 August 2021). The $PM_{2.5}$ and $PM_{10}$ mass concentrations were measured using an oscillating microbalance and/or β absorption. Concentrations of $SO_2$ were measured by ultraviolet (UV) fluorescence. The concentrations of CO and $O_3$ were measured using infrared and UV absorption, respectively. The concentrations of $NO_2$ were measured using a molybdenum converter and chemiluminescence. The CO surface mixing ratio dataset was obtained by the Measurements of Pollution in the Troposphere (MOPITT) instrument aboard NASA's Earth Observing System Terra spacecraft (https://asdc.larc.nasa.gov/data/MOPITT/MOP03JM.008/, last access: 4 December 2021). The CO emission inventory used in this study consisted of integrated datasets from a publicly available dataset of the Emissions Database for Global Atmospheric Research (EDGAR FOR HTAP V2, https://edgar.jrc.ec.europa.eu/dataset_htap_v2, last access: 6 September 2021) (Crippa et al., 2018; Chen et al., 2021b) and the Multi-resolution Emission Inventory for China (MEIC) from 2015 to 2020 (http://meicmodel.org/dataset-mix.html, last access: 3 December 2021) (Li et al., 2017b; Zheng et al., 2018b, 2021).

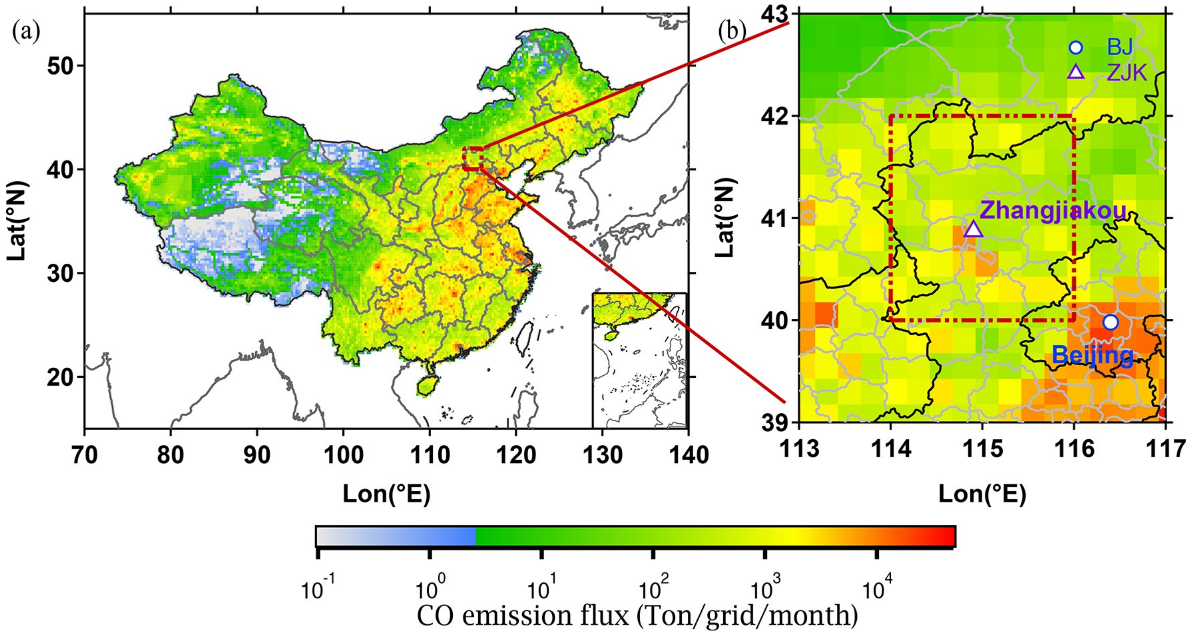

**Fig. 1.** (a) Emissions of CO in February in China during 2015–21. The red box denotes the geographical location of the simulation region; the map was taken from Igor software (©2021 Igor, https://www.wavemetrics.com, last access: 1 October 2021); (b) the geographical location of the simulation region and observation sites in BJ and ZJK.

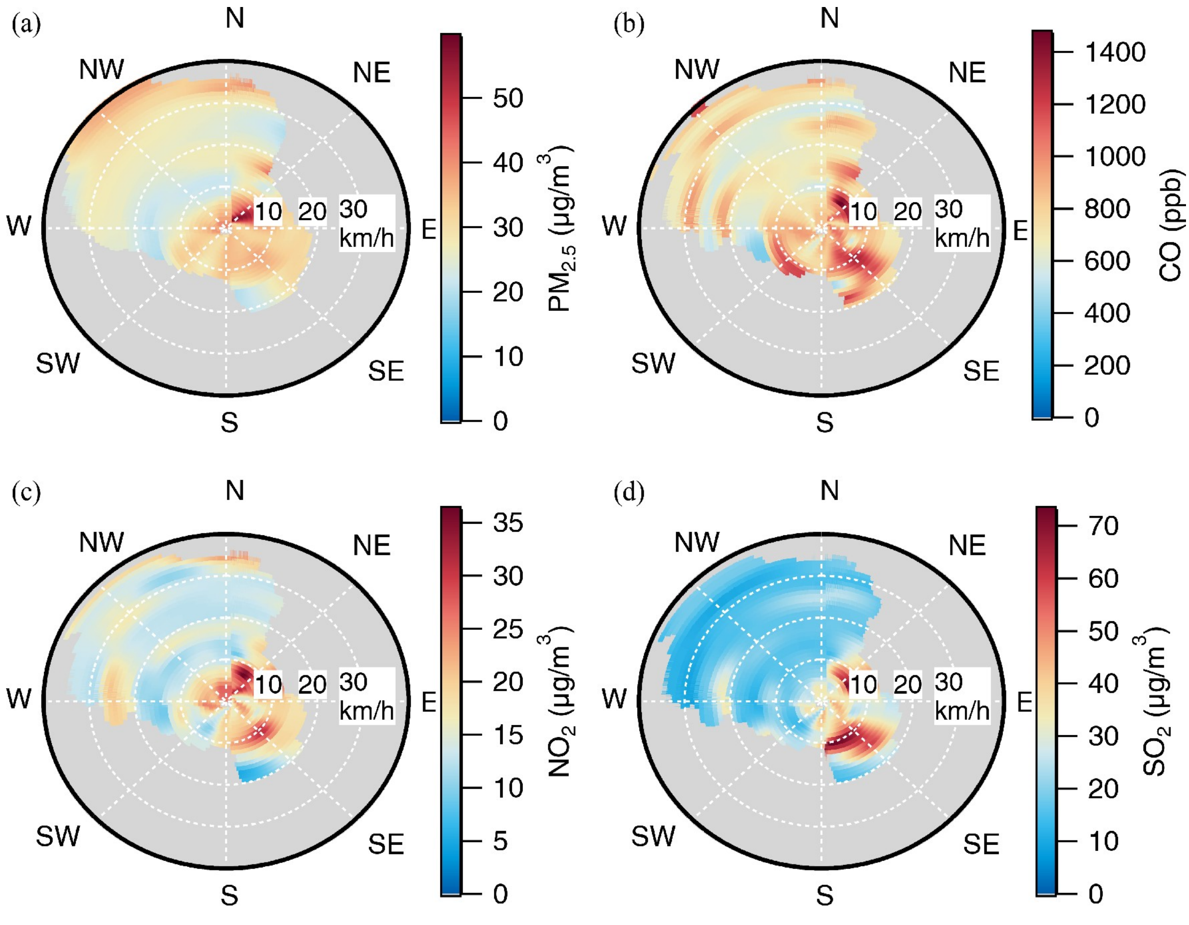

**Fig. 2.** Bivariate polar plots of (a) $PM_{2.5}$, (b) CO, (c) $NO_2$, and (d) $SO_2$ concentrations (μg $m^{-3}$) in February during 2015–21 in ZJK.

### 2.2. *Model calculation*

#### 2.2.1. *HYSPLIT model*

The HYbrid Single-Particle Lagrangian Integrated Trajectory Model (HYSPLIT), developed by the National Center for Environmental Prediction (NCEP) and National Center for Atmospheric Research (NCAR), was used to simulate the air footprint area of interest for the dispersion and trajectory analysis, which was based on the Lagrangian transport model (https://ready.arl.noaa.gov/HYSPLIT_traj.php, last access: 29 September 2021) (Stein et al., 2015; Sachdeva and Baksi, 2016). The potential uncertainty of the HYSPLIT model is related to the simulation settings and the meteorological assimilation datasets that drive the model simulation (Su et al., 2015). The HYSPLIT model provides the calculation of trajectory clustering based on many cluster analysis methods (Borge et al., 2007; Cabello et al., 2007; Baker, 2010; Karaca and Camci, 2010; Markou and Kassomenos, 2010), which can simplify the analysis and interpretation and reduce the uncertainty in the determination of atmospheric transport pathways. Trajectories are merged until the total spatial variance (TSV) of the individual trajectories with respect to their cluster-mean begins to increase dramatically (Stunder, 1996). The Global Data Assimilation System (GDAS) dataset (ftp://arlftp.arlhq.noaa.gov/pub/archives/gdas1, last access: 20 September 2021) was provided for HYSPLIT, which produces meteorological data four times a day at 0000, 0600, 1200, and 1800 UTC, and has a horizontal resolution of 2.5° × 2.5°. The vertical direction has 17 levels, ranging from the ground surface to 10 hPa. Meteorological parameters including wind, temperature, humidity, potential height, and ground precipitation are also provided. In this study, air parcels were released at 100 m above ground level from the Chongli District, ZJK (40°–42°N, 114°–116° E) (Fig. 1), and a 10–day backward trajectory was calculated for the period 2015–21 every six hours.

#### 2.2.2. *FLEXPART model*

In this study, the long-range transport and spatial distribution of the SRR were determined by a FLEXible PARTicle (FLEXPART) dispersion model developed by the Norwegian Institute for Air Research (https://www.flexpart.eu, last access: 5 September 2021). This model can simulate a large range of air parcel transport processes (Stohl et al., 2009), including tracing particles forward in time, from source areas, and backward in time, from given receptors. The SRR was expressed in units of seconds per kilogram in a particular grid cell and is proportional to the particle residence time in that cell, which is essentially used to simulate CO concentration at the receptor site in this study. Initial meteorological field data for FLEXPART are taken from NCEP FNL Operational Model Global Tropospheric Analysis data on a 1° × 1° grid, which provides global observation meteorological data at 0000, 0600, 1200, and 1800 UTC (https://rda.ucar.edu/datasets/ds083.2/#access, last access: 3 August 2021). During the simulation, one unit mass of particles, considered as an air sample, was released from Chongli District, ZJK (40°–42°N, 114°–116°E) (Fig. 1) at 100–500 m above ground level for 2015–2021. The SRR was calculated every three hours with a 10-day backward simulation. In this study, the residence time analysis (RTA, units: %) method is used to characterize the transport channels at the receptor sites (Ashbaugh et al., 1985), which is defined as:

$$\mathrm{RTA}\,(i,j) = \frac{t(i,j)}{T} \times 100\,. \qquad (1)$$

Here, $t(i,j)$ is the residence time of the particles in each cell outputted by the FLEXPART model, and $T$ is the sum of the residence time of all grid points.

## 3. Results and discussion

### 3.1. *Pollution characteristics in OWG locations*

#### 3.1.1. *Temporal variation of particle matter*

An atmospheric pollutant dataset during February from 2015 to 2021 was used to analyze the pollution characteristics of OWG locations during the Winter Olympics period. Figure 3 shows the monthly variations in $PM_{2.5}$, $PM_{10}$, CO, $NO_2$, and $SO_2$ during 2015–21. Although the ZJK particulate matter concentration was lower overall than in BJ, the trends in $PM_{2.5}$ concentrations in ZJK and BJ were different. There were two peaks in February 2017 and 2020, indicating that the impact of unfavorable meteorological conditions on OWG sites cannot be ignored (Fig. S1 in the Electronic Supplementary Material, ESM). The $PM_{2.5}$ concentrations in BJ and ZJK were the lowest in 2019 and 2018, with average concentrations of 34.1 and 23.7 μg $m^{-3}$, respectively. The $PM_{2.5}$ concentrations in BJ and ZJK in 2021 increased by 93% and 28% compared to those in 2019 and 2018, respectively. The $PM_{10}$ concentrations in BJ decreased dramatically in 2016 and showed an increasing trend after that. The $PM_{10}$ concentrations in ZJK showed a general downward trend from 2016 to 2020 and a 34% increase in 2021. The diurnal dynamics of the particle matter concentrations in BJ and ZJK are shown in Figs. 4a–d. The diurnal variation in $PM_{2.5}$ in Beijing was not significant. Yet, the daily variation in $PM_{2.5}$ in ZJK showed a clear U-shaped distribution, with the concentration decreasing at noon and increasing at 0800 and 1700 LST due to variations in mixing layer height and emissions.

#### 3.1.2. *Gaseous pollutants*

The monthly trends of CO in BJ and ZJK generally have the same tendency (Fig. 3), with large annual fluctuations in concentration and an overall decreasing trend, with the concentrations in 2021 decreasing by 41% and 25%, respectively, in both locations compared to 2015. The clean air action plans released by the state council contributed to CO reductions by regulating emissions and improving combustion efficiency in the steel and building materials industries, residential energy, and gasoline-powered cars. (Zheng et al., 2018b; Chu et al., 2020). Since 2017, the annual variation trend of $NO_2$ concentration in ZJK and BJ had initially

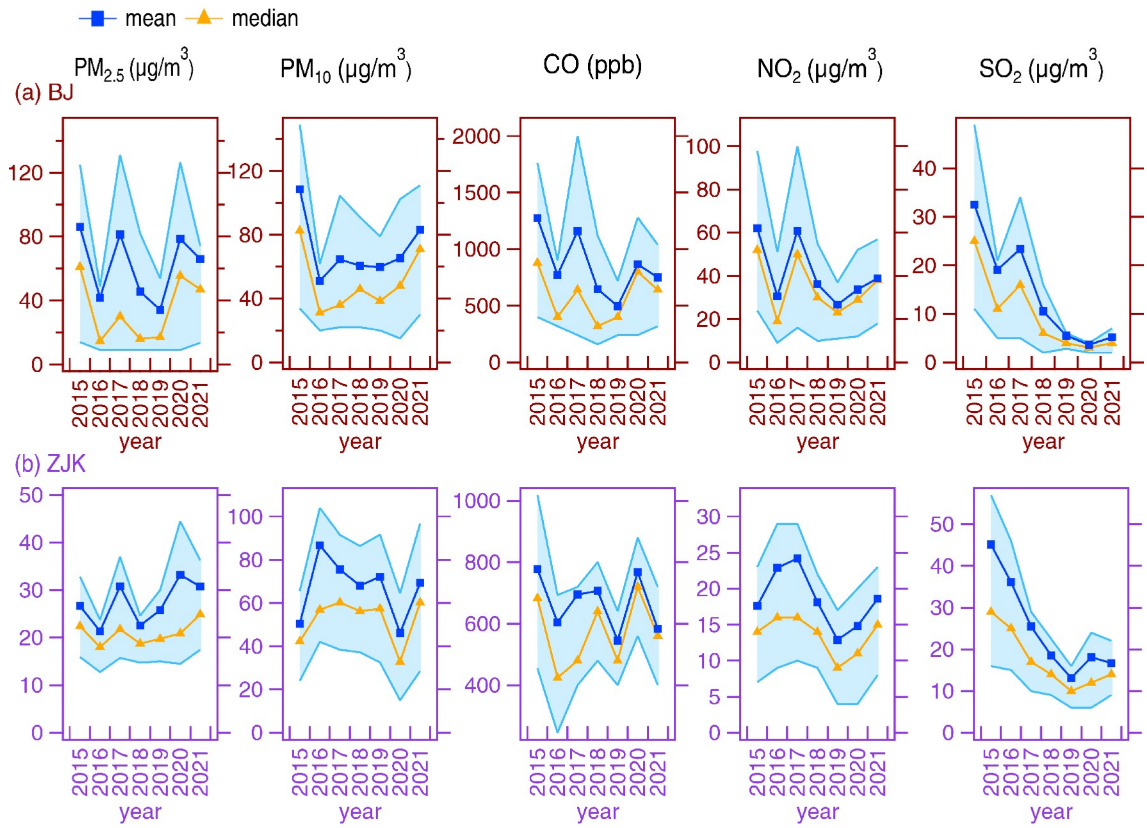

**Fig. 3.** Monthly variation in air quality indices ($PM_{2.5}$, $PM_{10}$, CO, $NO_2$, and $SO_2$) in (a) BJ and (b) ZJK from 2015 to 2021 during the XXIV OWG period. The orange triangles represent the median values; the blue squares represent the mean values; the upper and lower boundaries of the shaded areas represent the 75th and 25th percentiles of data points.

decreased before increasing and reached the lowest point in 2019. During the Winter Olympics, the design and implementation of vehicle control measures considered the increase in $NO_2$ concentration during recent years. The $SO_2$ concentration in BJ and ZJK in 2021 decreased by 84% and 63%, respectively, compared with 2015. This trend has been made possible by the abatement measures issued by the Chinese government in September 2013 and in June 2018, respectively, to reduce air pollutant emissions (Zheng et al., 2018a, b; Li et al., 2021a, b). The accelerated reduction in $SO_2$ is mainly due to the reduced emissions from coal combustion in the power and industrial sectors (Zhai et al., 2019; Li, 2020; Wang et al., 2021). Compared with the BJ site, the variation patterns of pollutants (such as $PM_{2.5}$ and $NO_2$) in the ZJK site have significant diurnal variation. Two $NO_2$ peaks at 0800 am and 1700 pm LST indicate a strong influence on traffic emissions.

#### 3.1.3. *Correlations of $PM_{2.5}$ and gaseous pollutants*

Unfavorable meteorological conditions and regional atmospheric transportation usually lead to a simultaneous increase in the concentrations of all atmospheric air pollutants, resulting in general correlations between $PM_{2.5}$ and gaseous pollutants. In this study, the Pearson correlation coefficients between $PM_{2.5}$ and gaseous pollutants at the BJ and ZJK stations for the 2015–21 period during the same period of the XXIV OWG were analyzed (Table S1 in the ESM). The most significant correlations between PM2.5 and CO were likely attributed to combustion activities. Almost all types of combustion emit CO and other gaseous precursors associated with $PM_{2.5}$ formations. The correlations between $PM_{2.5}$ and $NO_2$ suggest that traffic emissions sources make vital contributions to $PM_{2.5}$, as $NO_x$ is mainly from mobile sources (Yang et al., 2011; Chu et al., 2020). Furthermore, the correlations between $PM_{2.5}$ and $SO_2$ indicate that coal combustion from different sectors also contributes to $PM_{2.5}$. There is a negative relationship between $PM_{2.5}$ and $O_3$ in BJ and ZJK, which may be due to the reduction of fine particles that increase $O_3$ by reducing the heterogeneous $HO_2$ radical loss (Chu et al., 2020). Interestingly, the correlations between $PM_{2.5}$ and $O_3$ at BJ and ZJK have decreased over the past three years. One possible reason for this phenomenon is the effectiveness of the implemented emissions control measures.

### 3.2. *Atmospheric horizontal transport in OWG locations*

To investigate the potential emission source regions of pollutants in ZJK, the horizontal transport path of particles arriving at ZJK between 2015–21 in February was calculated using the FLEXPART model for 240 h and by using the RTA statistical method (Fig. 5). The value of the RTA at each grid point reflects the relative contribution of the different grid points to the ZJK. Prior to 2019, the RTA was high in northwest ZJK, and the RTA reached 0.1% in west-central Inner Mongolia and west-central Mongolia. During 2015–17, RTA values reached 0.01% or more in parts of Gansu and Ningxia Provinces east of the Qilian Mountains and north of the Qinling Mountains, as well as in southern

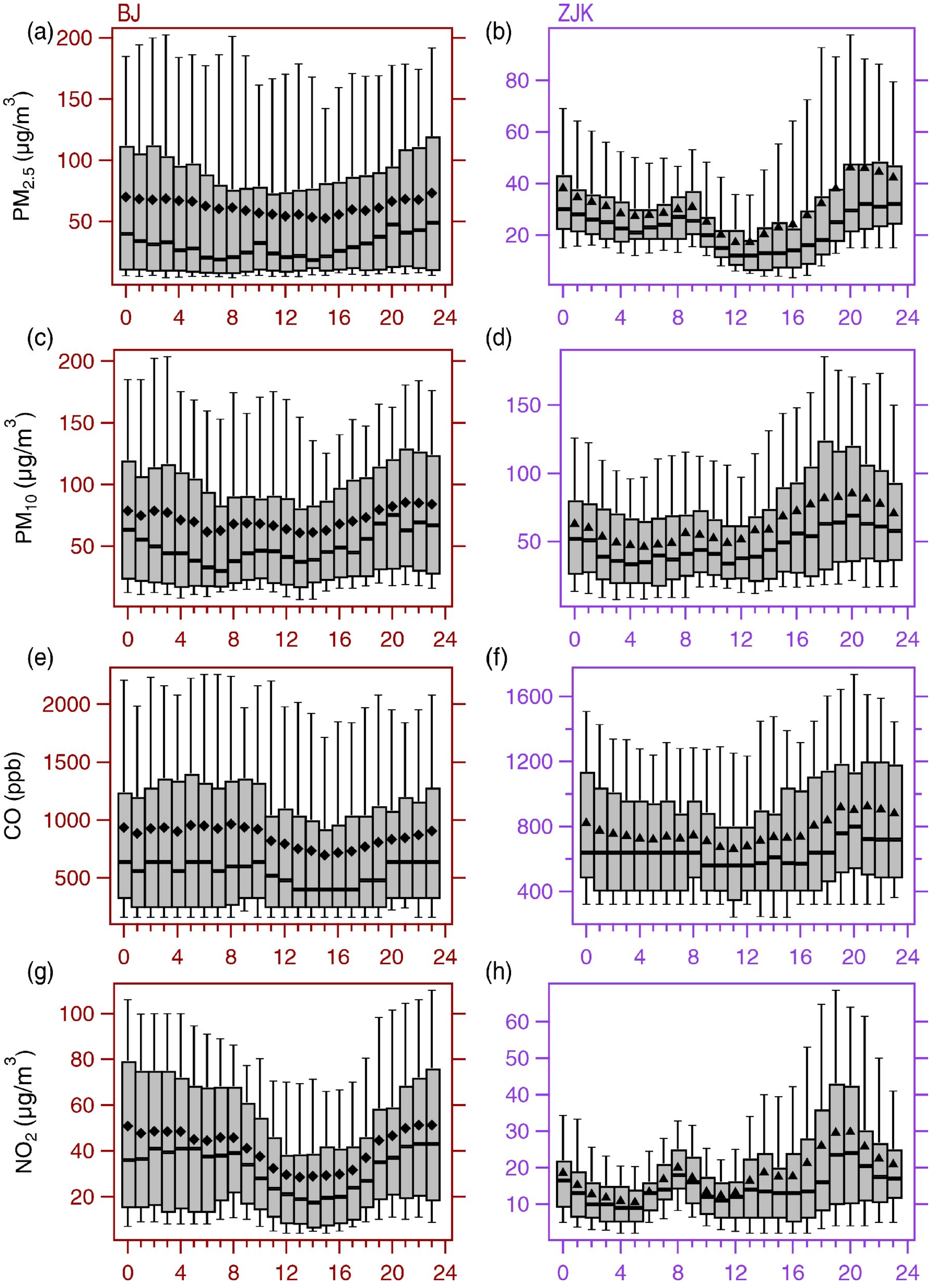

**Fig. 4.** Diurnal dynamics of $PM_{2.5}$, $PM_{10}$, CO, and $NO_2$ in BJ (left panel) and ZJK (right panel). The solid points represent the average values. The line inside the box indicates the median. The upper and lower boundaries of the box represent the 75th and 25th percentiles; the whiskers above and below each box represent the 90th and 10th percentile values.

Shaanxi and Xinjiang Provinces. This result indicates that potential source areas affecting air pollution in ZJK were mainly concentrated in the northwestern part of ZJK, such as central-eastern Inner Mongolia, western Inner Mongolia, and Mongolia, followed by Gansu, Ningxia, Xinjiang, and southern Shaanxi Provinces. There was a significant southward and westward shift in the RTA high-value area after 2019, and the highest RTA reached almost 1%. The main locations where the RTA reached 1% in the 2020–21 period were concentrated in ZJK and its southern regions. Regions

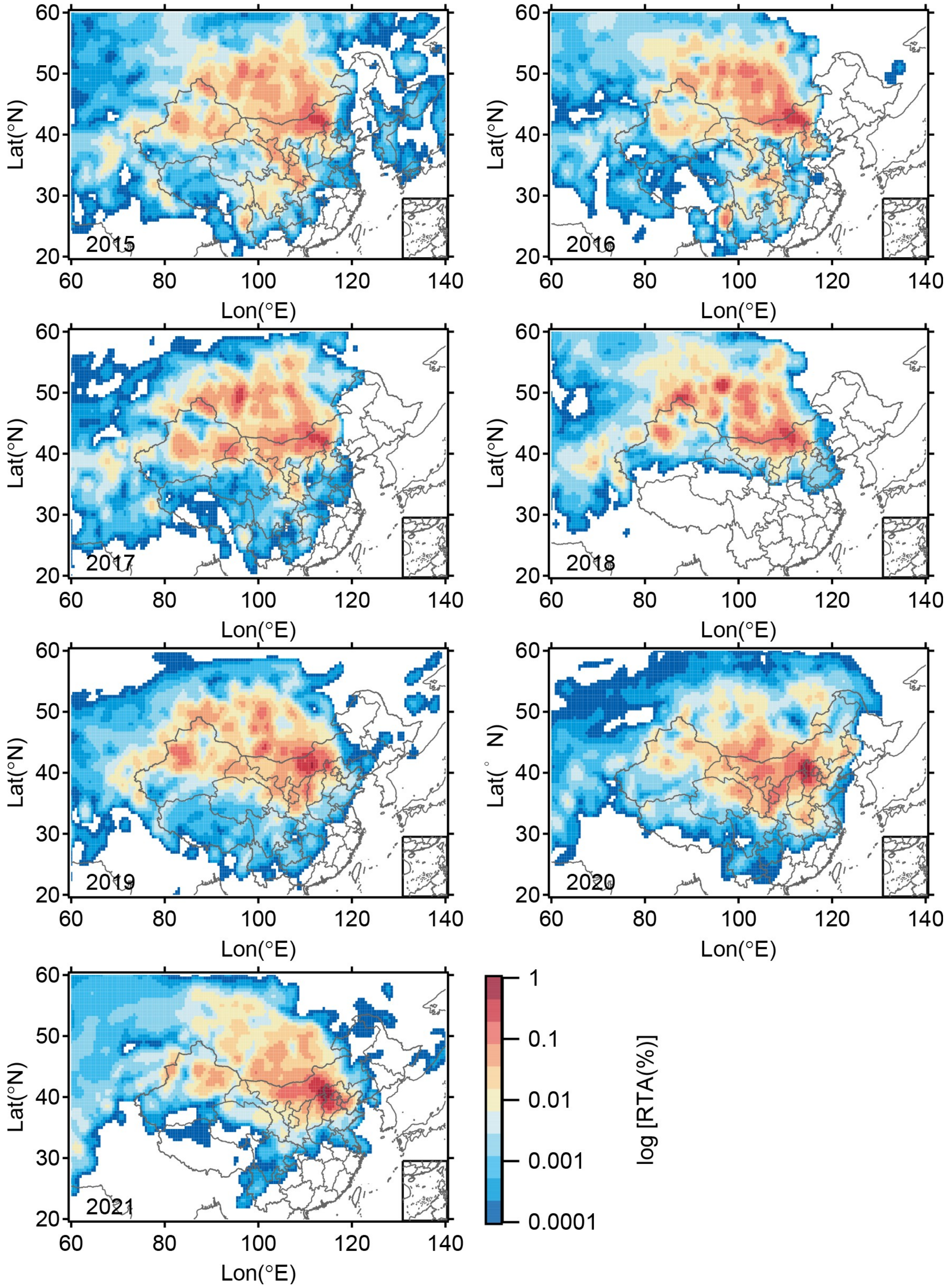

**Fig. 5.** Horizontal transport path of particles for 2015–21 for February using the FLEXPART model for 240 h.

with RTA values of 0.1% or more include southwestern Mongolia, central and western Inner Mongolia, and parts of Shaanxi and Shanxi Provinces. After 2019, the residence time of particulate matter in Zhangjiakou and its southern parts, central and western Inner Mongolia, Shaanxi, and Shanxi Provinces increased significantly compared to the period before 2019.

The variation anomaly of SRR can reflect the relative importance of different potential regions to the air pollutant in ZJK in different years. Using 2020 as an example, the SRR anomaly in 2020 was calculated by subtracting the SRR in 2020 by the average SRR over the 2015–21 period (Fig. 6). Before 2018, the SRR anomaly was positive in Inner Mongolia and Mongolia, which are northwest of ZJK, and negative in ZJK and its southern regions. The SRR anomaly transitioned to negative in Inner Mongolia and Mongolia after 2018 and positive in ZJK, south of ZJK, and in Shanxi and Shaanxi provinces west of ZJK after 2019. This result indicates that pollutant transport from northwestern ZJK had a stronger than average impact on ZJK. Conversely, transport from southern ZJK had a weaker than average impact on ZJK before 2018.

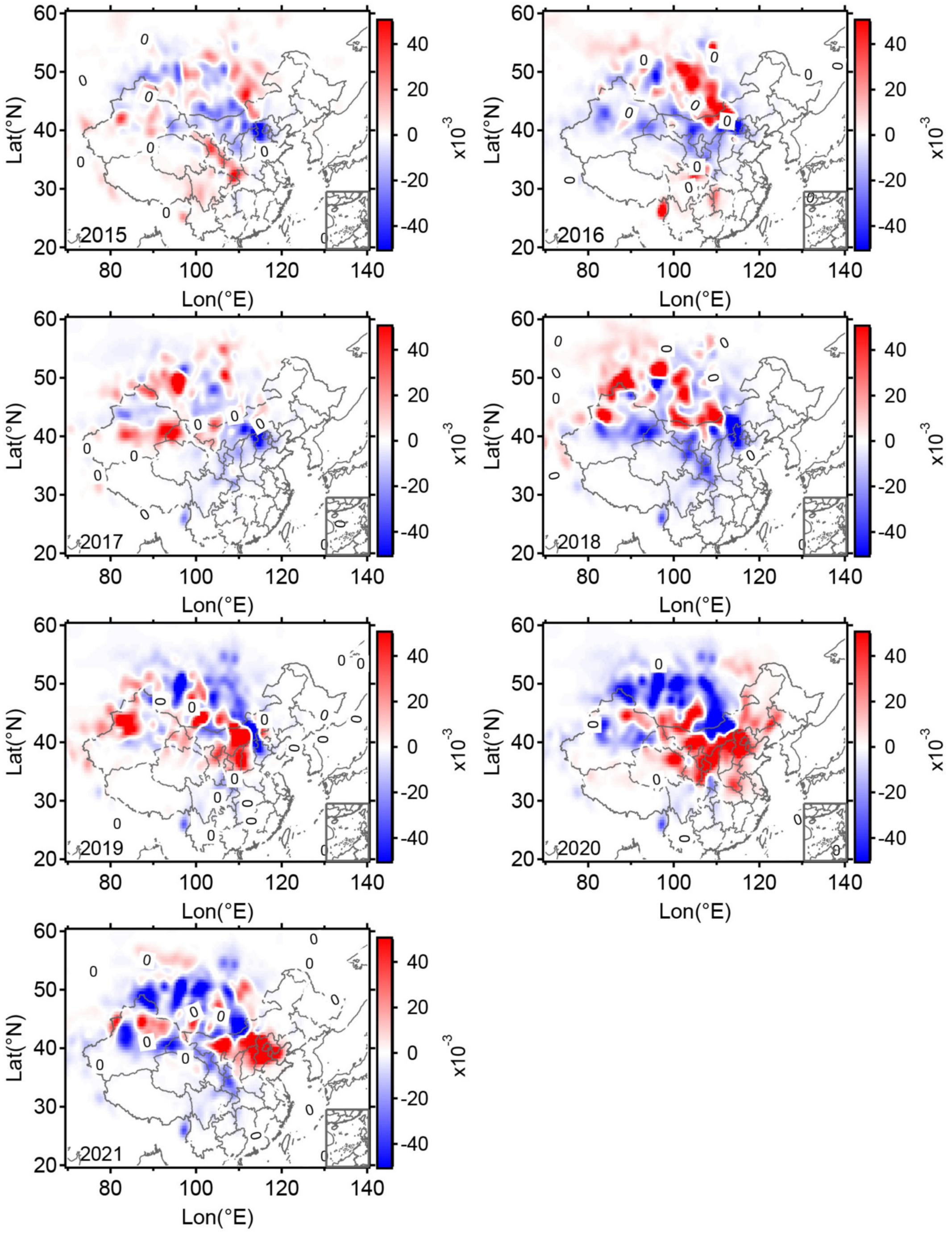

**Fig. 6.** The anomaly variations of the horizontal transport path of particles for 2015–21 for February (units: s).

#### 3.3. *Impact of atmospheric transport at different altitudes at OWG locations*

##### 3.3.1. *Influence of different air mass sources on pollutant concentrations*

The backward trajectories calculated by HYSPLIT for the periods 2015–21 were clustered. The clusters of air masses were determined from the change in TSV and the rate of change in TSV with a 30% criterion (Fig. S2). Five clusters of air masses were determined and renamed according to terrain and orientation (Fig. 7). Cluster 1 reached northwest of ZJK (NW) from the Mongolian Plateau (MP) and was characterized by slow air mass movement; we called it MP-NW1. Cluster 2, which reached ZJK via the Tianshan Mountains (TM) and the central Inner Mongolia city cluster (CIM), was named TM-CIM1. Cluster 3 flowed from the Arctic Ocean via the central Siberian Plateau (CSP) in northern Russia to the plateau in the north (NP) of ZJK and reached ZJK; this trajectory had a long transport path and was named CSP-NP. Cluster 4 reached northwest of ZJK (NW) from the Western Siberian Plateau (WSP) and was named WSP-NW2. Cluster 5, which was named KH-CIM2, arrived at ZJK via the Kazakh Hills (KH), MP, and CIM.

The relationship between the pollutants and air mass was also studied, as illustrated in Fig. 8. Among the five clusters, the highest $PM_{2.5}$ concentration, with an average concentration of 29.4 μg $m^{-3}$, appeared in the TM-CIM1 air mass, and the lowest mean $PM_{2.5}$ concentration, 20.3 μg $m^{-3}$, occurred in the CSP-NP air mass. The air mass of CSP-NP was mainly from the north with a relatively clean air type, while the TM-CIM1 air mass went through the CIM with relatively higher pollution. However, the influence of air masses on gaseous precursors was different from that of $PM_{2.5}$. The CO, $NO_2$, and $SO_2$ concentrations from KH-CIM2 were highest among the five clusters. The concentrations of $NO_2$ and $SO_2$ from CSP-NP were slightly lower than those from KH-CIM2. We found that the concentrations of gaseous precursors from the relatively clean air mass in the north were higher, and the concentrations of $PM_{2.5}$ were lower. In comparison, the concentrations of gaseous precur-

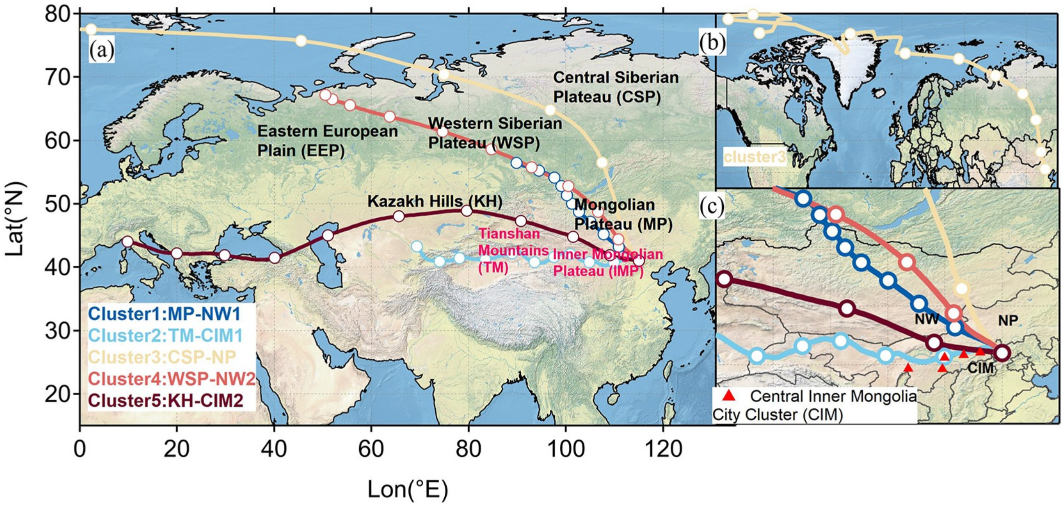

**Fig. 7.** (a) The five air mass clusters identified for the 2015–21 period, each represented by averaged 10-day backward trajectories; (b) the complete trajectory of Cluster 3; (c) the partially enlarged map of Zhangjiakou and its surrounding areas.

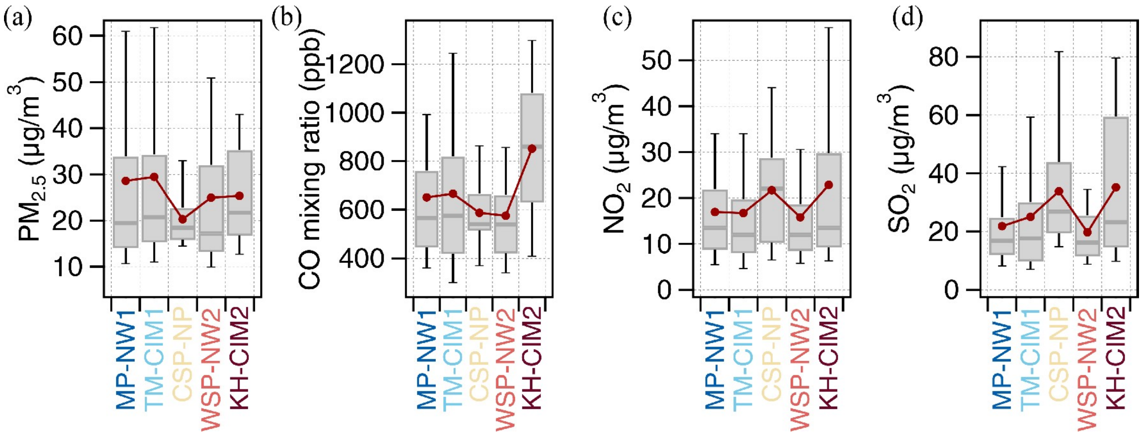

**Fig. 8.** The statistics of different pollutants ($PM_{2.5}$, CO, $NO_2$, $SO_2$) under distinct air masses.

sors and $PM_{2.5}$ were higher from the more polluted air mass in the west through CIM. This finding may indicate that secondary processes occurring in the air mass west of ZJK have a vital influence on ZJK.

#### 3.3.2. *The vertical distribution of atmospheric transport*

We focused our analysis on vertical transport based on air mass clusters, as illustrated in Fig. 9. Table S2 shows the height distribution of particle residence time for different backward time steps for the 2015–21 period. The percentage of RTA at $h \leqslant 1000$ m increased with time, while at $h > 1000$ m, the RTA percentage decreased with time among the five clusters. We suspected that the transport height of the particles decreased due to the weakening of the turbulent mixing effect near the release point after a certain time of the transport of the upper-level particles (Che et al., 2021). The high-value area of RTA was mainly concentrated at an altitude of < 200 m above ground level and during the backward hours of < 24 h. The high-value part of RTA from the two air masses of TM-CIM1 and KH-CIM2 is mainly concentrated in CIM, which is related to the obstruction of TM and MP. The air mass of CSP-NP had a long transport distance, but the high-value region of RTA was mainly concentrated below 600 m above the ground level of MP. In conclusion, the low-altitude transport of TM-CIM1 and KH-CIM2 has an important impact on the pollution of ZJK.

### 3.4. *Relative contribution of the different regions to the air quality of ZJK*

#### 3.4.1. *Source-Receptor model simulation*

The spatial distribution of SRR was determined by the FLEXPART Model (Fig. 10a). The mixing ratio of CO was simulated by the spatial integration of SRR and emission flux (Fig. 10b). A 10–day simulation period was demonstrated to be sufficient to explain most of the CO variability at the receptor, and a longer-day simulation would lead to increased model errors (Seibert and Frank, 2004; Pan et al., 2014). The output of FLEXPART includes 50 layers, and a footprint layer of 0–100 m was selected in the horizontal distribution and the calculation of the CO mixing ratio. Due to the long tropospheric CO lifetime, the mixing ratio at the observation sites was divided into two parts, the source area contribution based on SRR calculations and a superimposed regional background value. The CO mixing ratio of the regional background was determined by the minimum value of the CO mixing ratio time series at the monitoring locations. We compared the modeled CO mixing ratio at the receptor with the observed CO mixing ratio at the monitoring sites and the surface CO mixing ratio obtained from MOPITT (Figs. 10c and 11). The SRR-based simulation generally captured the main monthly variability of the CO mixing ratio in ZJK from 2015 to 2021. The Pearson correlation coefficient between the simulated and observed values reached ~ 0.94. The simulated CO mixing ratio values obtained by FLEXPART were relatively smaller than the observed values because of the underestimation of the CO emission inventory. The overall trends obtained by the simulation results show that the FLEXPART model is applicable to investigate the trends of air quality in ZJK.

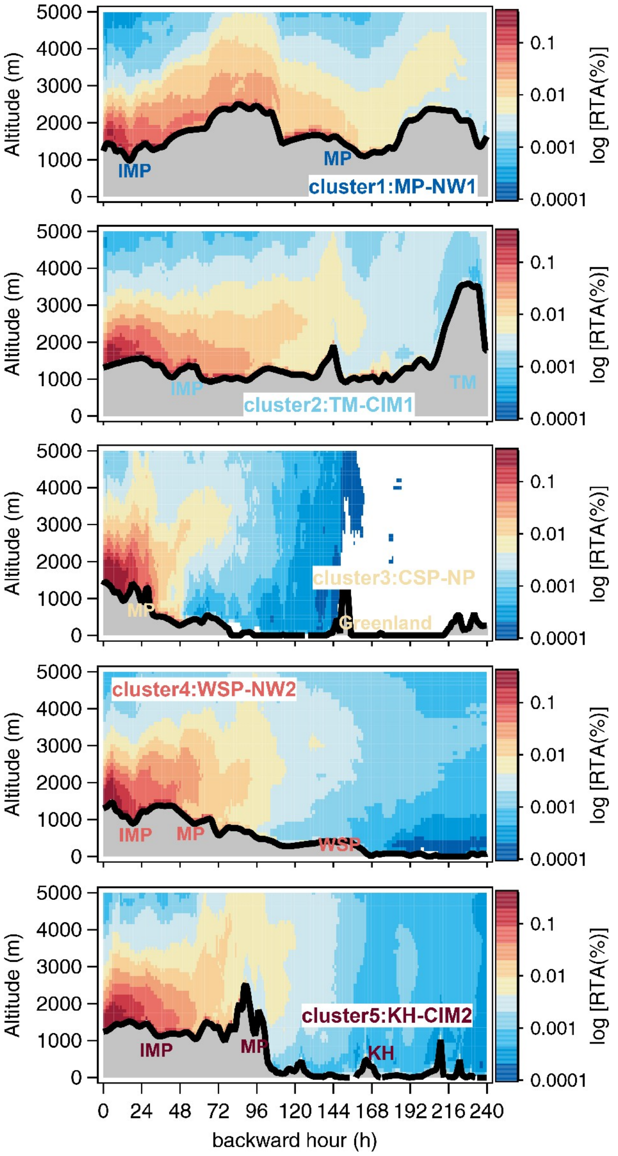

**Fig. 9.** Vertical transport path of particles for 2015–21 for February along the backward trajectories of different air masses.

#### 3.4.2. *Relative contributions*

Based on the above analysis, we selected four main potential areas with relatively larger impacts on ZJK, as shown in Fig. 10b; Inner Mongolia (IM), Gansu and Ningxia Provinces (GN), Fenwei Plain (FWP), and northern Shanxi Province and the North China Plain (NCPS) are marked with dark blue, sky blue, pink, and red colors, respectively. Figure 10d illustrates the relative contribution of four major regions to air pollution in ZJK for different years. Before 2019, ZJK was heavily influenced by IM, but after 2019, the relative contribution of IM to ZJK decreased, and the relative contribution of the NCPS to ZJK increased. We found a

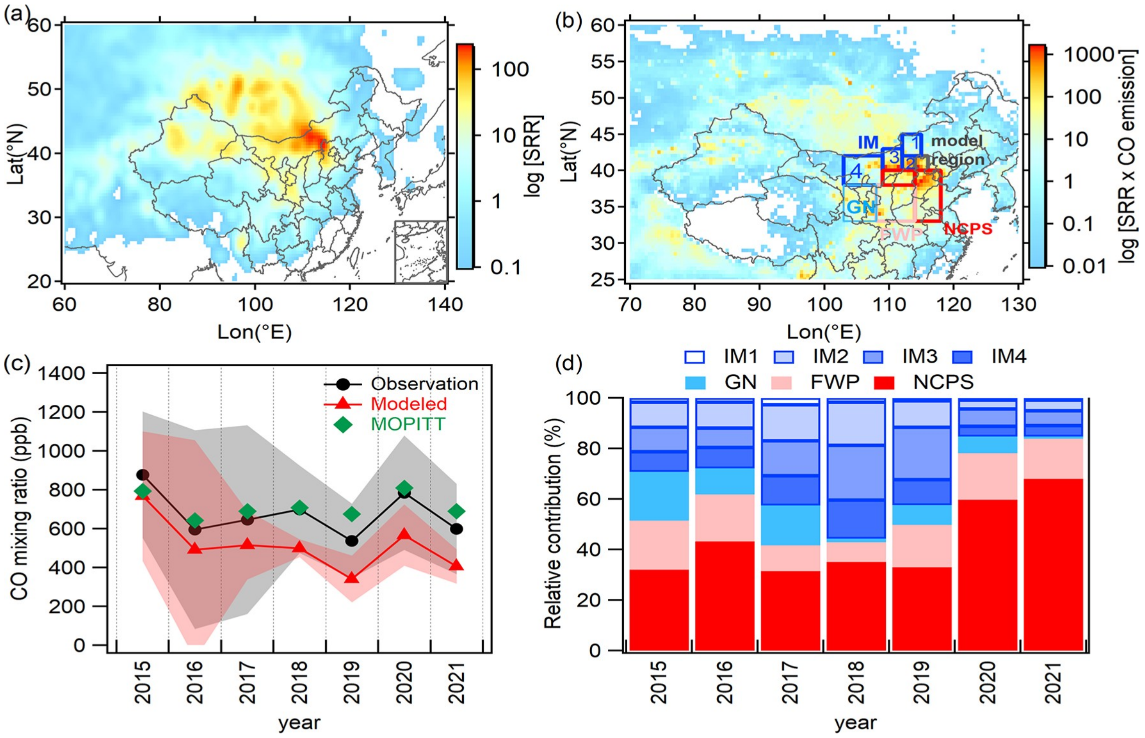

**Fig. 10.** (a) Source–receptor relationship; (b) relative contributions (arbitrary units) from potential source regions during 2015–21; (c) modeled, observed, and MOPITT CO mixing ratio in ZJK in February during 2015–21. The solid points represent the average values, and the error bars correspond to the standard deviations of the CO mixing ratio; (d) the relative contribution of different potential source areas to ZJK in different years.

larger relative contribution from central-IM in 2018, with increased CO concentrations in the ZJK region. This apparent shift is consistent with the results of the cluster analysis of air masses mentioned in section 3.3, where air masses passing through the central-IM city cluster transported higher concentrations of pollutant precursors. The relative contribution of NCPS to ZJK in 2021 was as high as 68%, an increase of 106% compared to 2019, corresponding to an increase in CO concentrations in ZJK from 2020 onward, which may be due to the increased relative contribution of the NCPS to air pollution in ZJK. As a corollary, the relative contribution of NCPS to air quality in ZJK in 2022 is still considerable based on observations and models. It is also necessary to strengthen the control of emissions from CIM cities, which also has a significant impact on ZJK under unfavorable meteorological conditions.

### 4. Concluding remarks and atmospheric implications

In this study, FLEXPART and HYSPLIT models were used to simulate the transport characteristics of atmospheric particulate matter in ZJK and its surrounding areas. The temporal variation in the air quality of the OWG was analyzed based on the air quality index dataset at each monitoring station in BJ and ZJK. The concentration of particulate matter in BJ was higher than that in ZJK. The $PM_{2.5}$ concentrations in BJ and ZJK increased after 2019 and 2018, respectively. The concentrations of $PM_{2.5}$ in 2021 increased by 93% and 28% compared to 2019 and 2018 in BJ and ZJK, respectively. The $PM_{10}$ concentrations in BJ decreased dramatically in 2016 and then trended upward, with concentrations decreasing by 23% in 2021 compared to 2015. The $PM_{10}$ concentrations in ZJK showed a general downward trend after 2016, with a 20% decrease in 2021 concentrations compared to 2016. The BJ and ZJK regions experienced significant decreases in CO and $SO_2$ concentrations over the last seven years, possibly due to the measures issued by the state council to reduce air pollution in September 2013 and June 2018, respectively. The increasing trend of $NO_2$ concentrations, which started in both BJ and ZJK after 2019, needs to be considered. Significant correlations were found between $PM_{2.5}$ and gaseous pollutants (CO, $NO_2$, and $SO_2$) over the past seven years in BJ and ZJK. This suggested that vehicle emissions and coal combustion were important contributors to $PM_{2.5}$. In BJ and ZJK, the $PM_{2.5}$ and $O_3$ were inversely related, which may be because of the reduction of particles increasing $O_3$. The correlation between $PM_{2.5}$ and $O_3$ in both BJ and ZJK has decreased over the last three years due to emission control measures.

The analysis based on the SRR calculated by FLEXPART leads to the following conclusions. The horizontal transport path has changed significantly in the last seven years. Before 2018, the air masses from Inner Mongolia and Mongolia mainly affected ZJK; after 2018, the air mass transport from west and south of ZJK, such as Shanxi Province

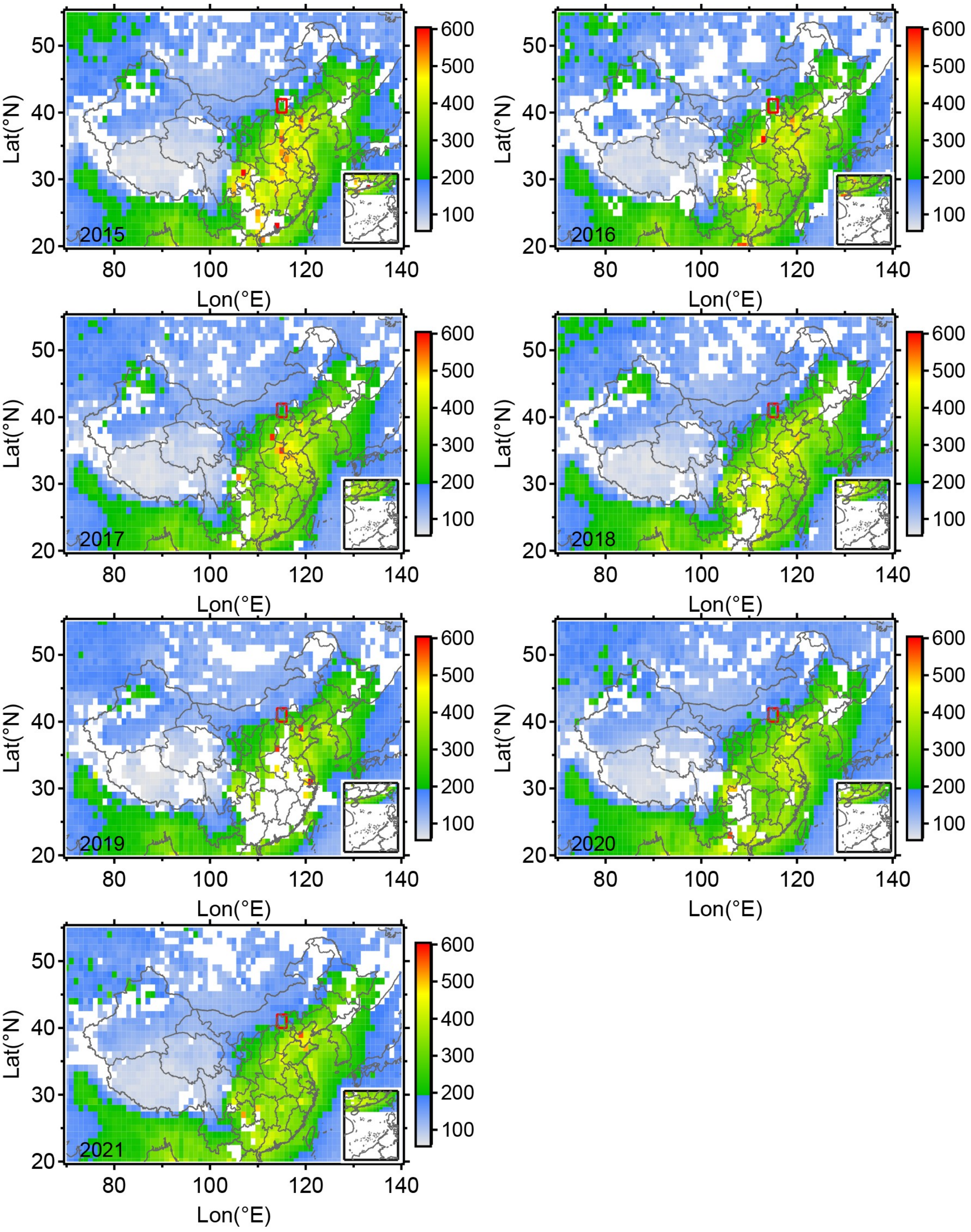

**Fig. 11.** The surface CO mixing ratio as measured by the MOPITT instrument flying aboard NASA's Earth Observing System Terra spacecraft for the February months of 2015–21 (unit: ppb). The red box represents the FLEXPART model region.

and the NCP, strengthened. The 10-day backward trajectories were grouped into five clusters during the February months of 2015–21. The air masses of TM-CIM1 and KH-CIM2 were characterized by relatively high concentrations of gaseous precursors and $PM_{2.5}$, followed by MP-NW1 and WSP-NW2. The air from the cluster of CSP-NP was clean. The transport height of the particles decreased within 72 h of approaching ZJK, and the transport altitude of each air mass cluster was mainly concentrated below an altitude of 600 m above ground level.

The variation in the mixing ratio of CO calculated by the FLEXPART model was largely consistent with the observed CO, which indicates that the source-receptor model simulation is suitable for investigating air quality in

ZJK. Based on the SRR and emission inventory, the relative contribution of NCPS to air pollution in ZJK increased from 2020, consistent with the rebound in the CO mixing ratio. Additionally, central-IM significantly impacts ZJK under unfavorable meteorological conditions, which indicates that monitoring air pollution and emissions in the NCPS and central-IM should be strengthened during the XXIV OWG.

***Acknowledgements***. We thank the National Key Scientific and Technological Infrastructure project "Earth System Science Numerical Simulator Facility" (EarthLab).

***Open Access*** This article is licensed under a Creative Commons Attribution 4.0 International License, which permits use, sharing, adaptation, distribution and reproduction in any medium or format, as long as you give appropriate credit to the original author(s) and the source, provide a link to the Creative Commons licence, and indicate if changes were made. The images or other third party material in this article are included in the article's Creative Commons licence, unless indicated otherwise in a credit line to the material. If material is not included in the article's Creative Commons licence and your intended use is not permitted by statutory regulation or exceeds the permitted use, you will need to obtain permission directly from the copyright holder. To view a copy of this licence, visit http://creativecommons.org/licenses/by/4.0/.

***Electronic supplementary material:*** Supplementary material is available in the online version of this article at https://doi.org/10.1007/s00376-022-1463-1.

## Electronic Supplementary Material to

Transport Patterns and Potential Sources of Atmospheric Pollution during the XXIV Olympic Winter Games Period
