# Supplementary material for: Transport Patterns and Potential Sources of Atmospheric Pollution during the XXIV Olympic Winter Games Period
Source: Adv Atmos Sci. 2022 Apr 6;39(10):1608–22. doi: 10.1007/s00376-022-1463-1 (PMC8983323; doi:10.1007/s00376-022-1463-1)
Supplement: Supplementary file 1 — Transport Patterns and Potential Sources of Atmospheric Pollution during the XXIV Olympic Winter Games Period [file 376_2022_1463_MOESM1_ESM.pdf]

Electronic Supplementary Material to:
Transport Patterns and Potential Sources of Atmospheric Pollution during the XXIV Olympic Winter Games Period*

Yuting ZHANG[1,2], Xiaole PAN[1], Yu TIAN[1], Hang LIU[1], Xueshun CHEN[1,3], Baozhu GE[1,3], Zhe WANG[1], Xiao TANG[1], Shandong LEI[1,2], Weijie YAO[1,2], Yuanzhe REN[4], Yongli TIAN[4], Jie LI[1], Pingqing FU[5], Jinyuan XIN[1,2,6], Yele SUN[1,2,3], Junji CAO[7], and Zifa WANG[1,2,3]

[1]*State Key Laboratory of Atmospheric Boundary Layer Physics and Atmospheric Chemistry*,
Institute of Atmospheric Physics, *Chinese Academy of Sciences*, *Beijing* 100029, *China*
[2]*College of Earth and Planetary Sciences*, *University of Chinese Academy of Sciences*, *Beijing* 100049, *China*
[3]*Center for Excellence in Regional Atmospheric Environment*, *Institute of Urban Environment*,
Chinese Academy of Sciences, *Xiamen* 361021, *China*
[4]*Inner Mongolia Autonomous Region environmental monitoring central station*, *Hohhot* 010090, *China*
[5]*Institute of Surface-Earth System Science*, *Tianjin University*, *Tianjin* 300072, *China*
[6]*Collaborative Innovation Center on Forecast and Evaluation of Meteorological Disasters*,
Nanjing University of Information Science and Technology, *Nanjing* 210044, *China*
[7]*Institute of Atmospheric Physics*, *Chinese Academy of Sciences*, *Beijing* 100029, *China*

ESM to: Zhang, Y. T., and Coauthors, 2022: Transport patterns and potential sources of atmospheric pollution during the XXIV Olympic Winter Games period. *Adv. Atmos. Sci.*, **39**(10), 1608−1610, https://doi.org/10.1007/s00376-022-1463-1.

Table S1. The Pearson correlation coefficients between $PM_{2.5}$ and gaseous pollutants for the ATZX and BBF stations during 2015–21 for the same period of the XXIV Olympic Winter Games.

	BJ				ZJK			
Year	CO	NO_2	SO_2	O_3	CO	NO_2	SO_2	O_3
2015	0.90	0.80	0.85	–0.64	0.28	0.17	0.06	–0.51
2016	0.88	0.74	0.71	–0.65	0.92	0.84	0.62	–0.58
2017	0.93	0.89	0.84	–0.59	0.96	0.90	0.82	–0.58
2018	0.89	0.64	0.82	–0.37	0.82	0.79	0.72	–0.63
2019	0.79	0.53	0.49	–0.45	0.63	0.58	0.43	–0.35
2020	0.94	0.70	0.34	–0.18	0.71	0.78	0.42	–0.05
2021	0.88	0.35	0.53	–0.10	0.64	0.64	0.48	–0.33

*The online version of this article can be found at https://doi.org/10.1007/s00376-022-1463-1.

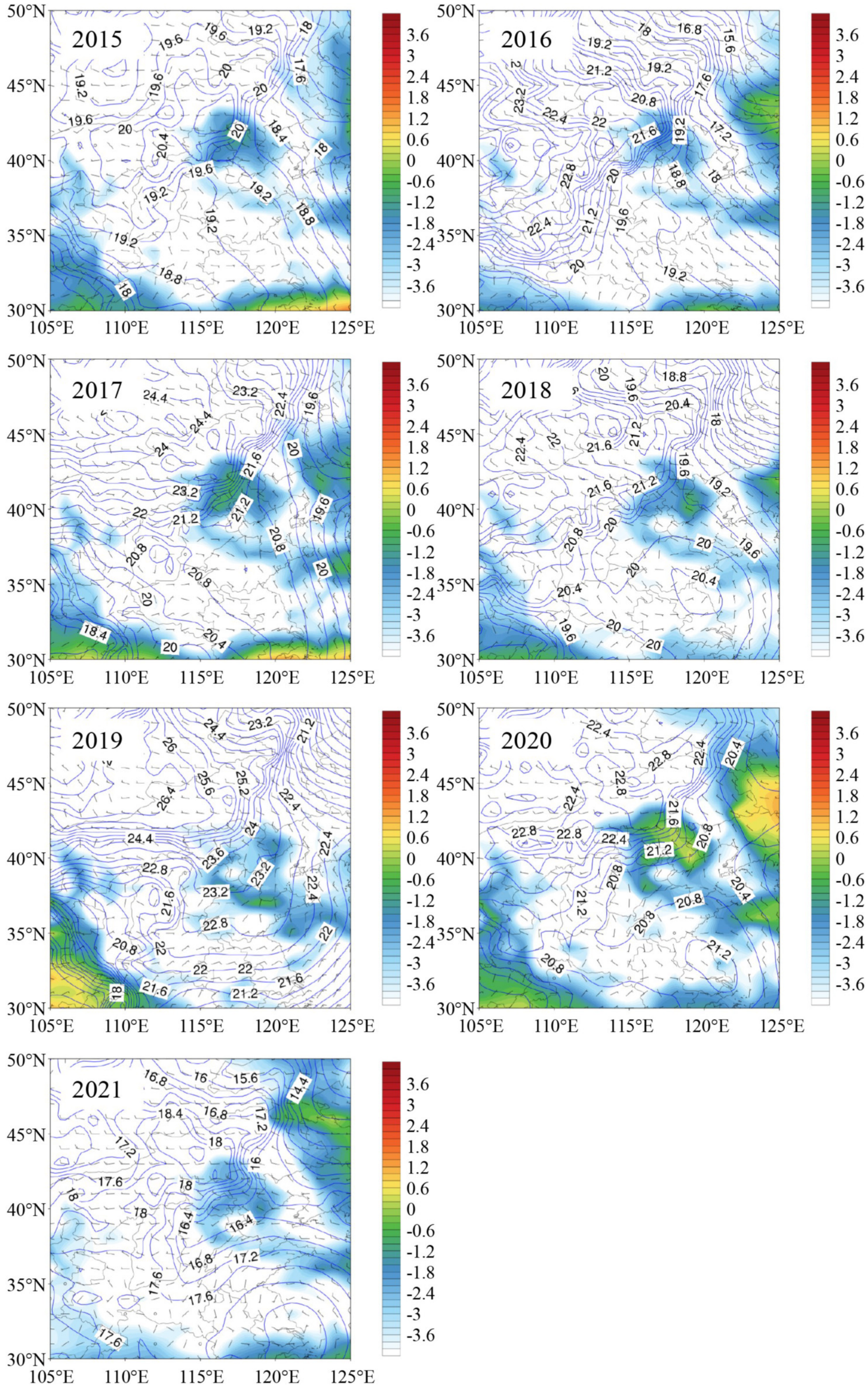

Fig. S1. 1000 hPa wind field (units: m s^{-1}) and geopotential height field (units: 10^{-1}gpm), the shaded areas represent the temperature differences between 900 hPa and 1000 hPa (units: °C) in February from 2015 to 2021.

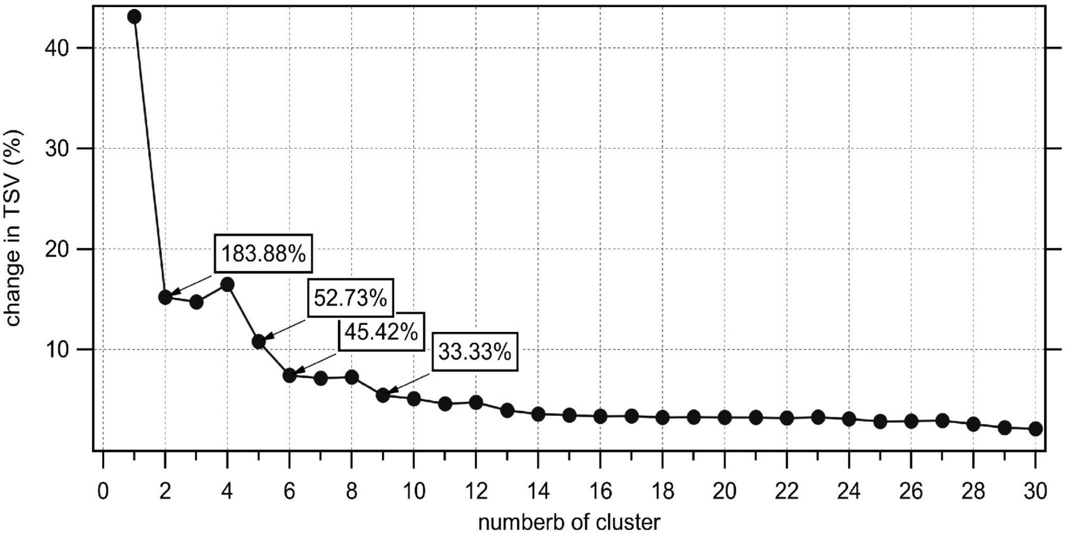

Fig. S2. The standard change in total spatial variance (TSV) as clusters are combined during 2015–21.

Table S2. Height distribution of RTA with different backward time steps during 2015–21 (units: %).

Cluster	Height (units: m)	24 h	48 h	72 h	96 h	120 h	144 h	168 h	192 h	216 h	240 h
C1	$0 < h \leqslant 200$	36.6	32.7	14.6	21.0	27.7	37.7	19.3	9.0	4.9	4.5
	$200 < h \leqslant 400$	22.2	14.4	19.5	10.3	9.1	8.7	10.6	6.1	4.6	3.5
	$400 < h \leqslant 600$	16.5	12.3	14.9	9.8	9.3	7.8	9.3	6.8	5.5	4.6
	$600 < h \leqslant 1000$	15.6	19.4	19.2	18.7	17.8	14.6	17.0	14.8	12.4	9.5
	$1000 < h \leqslant 2000$	7.3	17.3	24.1	27.5	24.3	19.9	26.4	31.3	30.6	26.5
	$2000 < h \leqslant 5000$	1.7	4.0	7.7	12.7	11.8	11.3	17.4	32.0	42.0	51.5
C2	$0 < h \leqslant 200$	35.3	30.7	29.6	21.7	10.8	11.5	14.2	20.1	8.0	3.6
	$200 < h \leqslant 400$	23.8	14.5	12.8	11.3	7.3	5.1	3.6	6.2	3.8	2.0
	$400 < h \leqslant 600$	16.8	13.6	10.6	10.0	8.3	5.1	3.8	4.8	3.7	2.0
	$600 < h \leqslant 1000$	14.6	18.9	16.9	17.2	17.4	11.9	9.0	7.4	8.0	5.1
	$1000 < h \leqslant 2000$	7.5	16.4	20.4	25.0	34.4	31.4	29.0	19.3	20.4	19.9
	$2000 < h \leqslant 5000$	2.0	5.8	9.8	14.8	21.7	35.0	40.4	42.3	56.2	67.3
C3	$0 < h \leqslant 200$	32.0	18.6	3.2	1.9	2.1	3.8	2.6	1.6	1.4	1.0
	$200 < h \leqslant 400$	22.9	9.4	1.1	1.2	1.4	3.1	1.7	2.0	0.6	0.6
	$400 < h \leqslant 600$	17.3	9.0	1.4	1.7	1.5	3.1	1.6	2.5	0.5	0.5
	$600 < h \leqslant 1000$	16.6	16.0	4.1	4.2	3.7	5.5	3.2	4.9	1.4	1.2
	$1000 < h \leqslant 2000$	8.7	27.0	18.7	17.1	16.9	15.7	10.5	12.4	5.4	3.9
	$2000 < h \leqslant 5000$	2.6	20.1	71.6	73.9	74.3	68.8	80.4	76.6	90.7	92.8
C4	$0 < h \leqslant 200$	36.6	26.6	15.5	7.5	4.0	3.6	2.9	1.3	0.5	1.0
	$200 < h \leqslant 400$	20.7	13.9	5.9	4.9	2.6	2.2	2.3	1.4	0.8	1.0
	$400 < h \leqslant 600$	15.9	12.1	6.7	5.7	3.5	2.6	2.2	1.8	1.4	1.6
	$600 < h \leqslant 1000$	16.3	19.0	16.3	12.6	8.7	6.3	5.3	4.9	4.0	4.3
	$1000 < h \leqslant 2000$	8.4	21.3	36.0	30.2	25.3	20.6	20.9	21.1	20.3	17.6
	$2000 < h \leqslant 5000$	2.0	7.1	19.8	39.0	56.1	64.7	66.4	69.5	72.9	74.6
C5	$0 < h \leqslant 200$	34.7	27.2	17.5	22.6	2.0	1.4	6.1	7.7	9.4	8.3
	$200 < h \leqslant 400$	23.8	15.0	9.1	4.7	2.0	1.5	2.5	2.9	2.7	2.8
	$400 < h \leqslant 600$	16.4	13.1	9.3	3.8	2.6	2.0	2.6	3.1	2.9	2.7
	$600 < h \leqslant 1000$	14.5	20.0	16.8	7.1	6.6	5.3	5.7	7.3	6.4	5.9
	$1000 < h \leqslant 2000$	8.2	18.8	27.4	19.2	23.5	18.9	18.1	19.7	18.9	17.8
	$2000 < h \leqslant 5000$	2.4	5.8	19.9	42.6	63.3	70.9	65.0	59.3	59.6	62.5
